# Comparison of Postoperative Coronal Leg Alignment in Customized Individually Made and Conventional Total Knee Arthroplasty

**DOI:** 10.3390/jpm11060549

**Published:** 2021-06-12

**Authors:** Felix Wunderlich, Maheen Azad, Ruben Westphal, Thomas Klonschinski, Patrick Belikan, Philipp Drees, Lukas Eckhard

**Affiliations:** 1 Department of Orthopedics and Traumatology, University Medical Center of the Johannes Gutenberg University Mainz, 55131 Mainz, Germany; thomas.klonschinski@unimedizin-mainz.de (T.K.); patrick.belikan@unimedizin-mainz.de (P.B.); philipp.drees@unimedizin-mainz.de (P.D.); lukas.eckhard@unimedizin-mainz.de (L.E.); 2 Clinic for Traumatology and Orthopedics, Heilig-Geist-Hospital Bingen, 55411 Bingen, Germany; maheen.azad@marienhaus.de; 3 Institute of Medical Biostatistics, Epidemiology and Informatics, University Medical Center of the Johannes Gutenberg University Mainz, 55131 Mainz, Germany; ruben.westphal@unimedizin-mainz.de

**Keywords:** total knee arthroplasty, leg alignment, patient-specific instruments, custom-made implant, rotational correction

## Abstract

Neutral coronal leg alignment is known to be important for postoperative outcome in total knee arthroplasty (TKA). Customized individually made implants (CIM) instrumented with patient-specific cutting guides are an innovation aiming to increase the precision and reliability of implant positioning and reconstruction of leg alignment. We aimed to compare reconstruction of the hip–knee–ankle angle (HKA) of the novel CIM system iTotal™ CR G2 (ConforMIS Inc.) to a matched cohort of the off-the-shelf (OTS) knee replacement system Vanguard™ CR (Zimmer Biomet). Retrospective analysis of postoperative coronal full-leg weight-bearing radiographs of 562 TKA (283 CIM TKA, 279 OTS TKA) was conducted. Via a medical planning software, HKA and rotation of the leg were measured in postoperative radiographs. HKA was then adjusted for rotational error, and 180° ± 3° varus/valgus was defined as the target zone HKA. Corrected postoperative HKA in the CIM group was 179.0° ± 2.8° and 179.2° ± 3.1° in the OTS group (*p* = 0.34). The rate of outliers, outside of the ±3° target zone, was equal in both groups (32.9%). Our analysis showed that TKA using patient-specific cutting guides and implants and OTS TKA implanted with conventional instrumentation resulted in equally satisfying restoration of the coronal leg alignment with less scattering in the CIM group.

## 1. Introduction

Total knee arthroplasty is a common and reliable procedure for successfully treating end-stage osteoarthritis (OA) of the knee. Although continued development of implant design, surgical technique, and postoperative follow-up treatment has improved the overall outcome of the procedure, there is still a noticeable number of patients who remain partially unsatisfied after TKA [1]. Amongst other factors, correct fitting and position of the TKA components with consecutive restoration of the axial alignment and mechanical axis of the limb lead to a good postoperative outcome and longer implant survival [2,3,4,5]. To maximize the capabilities of TKA regarding these factors, patient-specific customized implants have been developed in the recent past [6,7]. One of these implants is the patient-specific cruciate retaining knee replacement system iTotal™ CR G2 with custom-made implants and instruments, using computer-aided design and manufacturing (CAD/CAM) based on computed tomography (CT) scans of the patients’ leg. The goal of this implant is to restore a neutral postoperative mechanical axis, reduce bone resection, and optimize component fit. Previously published results are promising [8,9], although studies comparing CIM TKA to off-the-shelf implants implanted using conventional instrumentation are scarce, while most existing studies focus on patient-specific instrumentation rather than patient-specific implants. We therefore aimed to compare restoration of the hip–knee–ankle angle of the novel patient-specific knee replacement system iTotal CR G2 (ConforMIS Inc.; Burlington, MA, USA) to a matched cohort of the traditional knee replacement system Vanguard™ CR (Zimmer Biomet; Warsaw, IN, USA).

## 2. Materials and Methods

In total, 562 patients undergoing TKA (right: 235; left: 205; bilateral: 122) were included in the retrospective analysis with a distribution of 283 patient-specific knee replacement systems, iTotal™ CR G2, and a matched cohort of 279 traditional knee replacement systems, Vanguard™ CR. Both products match the country product clearances for Germany and are approved by the United States Food and Drug Administration (FDA).

All surgeries were conducted from 2015 to 2020 by the endoprosthetics team of the Department of Orthopedics and Traumatology of the University Medical Center of the Johannes Gutenberg University, containing four primary surgeons. Indication for TKA was end-stage primary or posttraumatic OA of the knee with no signs of ligamentous instability. Patients with varus or valgus deformity >15° were excluded due to eligibility criteria of the implants. For preoperative planning, all patients received coronal full-leg weight-bearing radiographs as well as antero-posterior lateral, and patella tangential conventional radiographs of the affected knee. Planning of the OTS Vanguard™ CR system was conducted via the mediCAD 2D Knee planning software (mediCAD Hectec GmbH, Altdorf, BY, Germany). In the case of a planned implantation of the iTotal™ CR G2 system, a CT-scan of the affected leg was conducted with a standard protocol and the CIM was designed and manufactured using the iFit software algorithm and 3D CAD/CAM technology as previously described by Arnholdt et al. [8]. We used a standard midline incision and medial parapatellar capsulotomy in all patients, adding local infiltration analgesia containing ropivacaine and adrenalin as well as i.v. and intraarticular tranexamic acid at the end of each surgery. No tourniquet or drainage was used. Postoperative radiological control of implant fit and leg axis was conducted via ap and lateral knee radiographs and coronal full-leg weight-bearing radiographs as soon as the patient was able to walk stairs and a full extension of the operated knee was possible.

**Figure 1:**
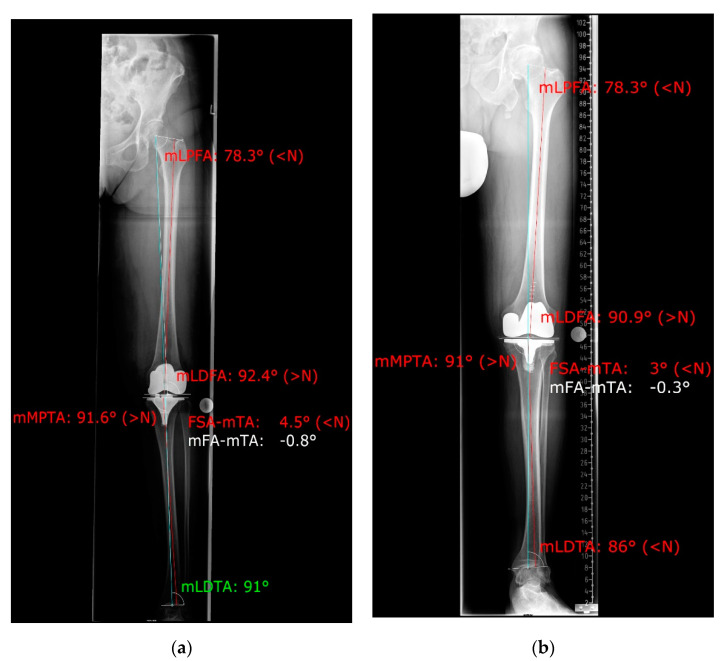
Measuring the HKA angle in mediCAD 2D planning software (**a**) iTotal CR G2 patient specific implant; (**b**) Vanguard CR conventional implant.

**Figure 2:**
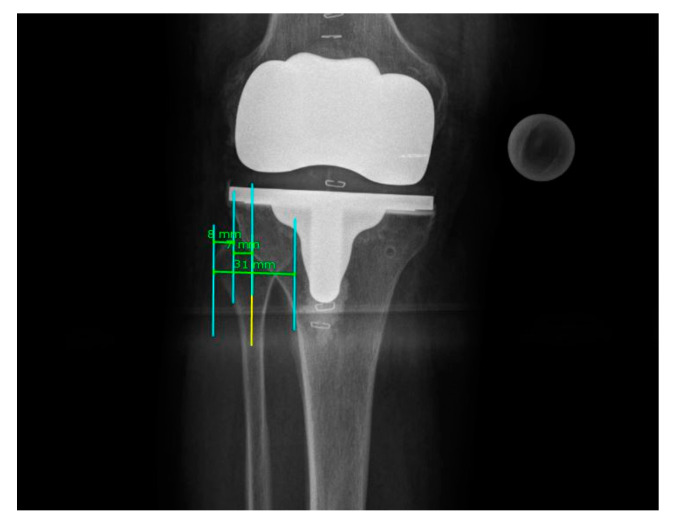
Detail of rotational analysis of a full-leg weight-bearing radiograph using the proximal tibio-fibular overlap.

Radiographic analysis of the postoperative coronal leg alignment was executed using the mediCAD 2D planning software on postoperative coronal full-leg weight-bearing radiographs. The radiographs were first checked for eligibility according to the following quality criteria: missing postoperative pictures, minor quality with incomplete imaging of the operated leg or poor image quality, and excessive rotational error. For determination of the leg axis, the HKA was measured using the angle between the mechanical axis of the femur (FMA) and tibia (TMA) (Figure 1). The operation aimed to restore a neutral mechanical alignment (180° ± 3° varus/valgus). For further improvement of the measurement accuracy, we calculated rotational correction for the measured HKA using the formula published by Maderbacher et al. in 2014 and 2021 [10,11], which is based on the proximal tibio-fibular overlap in long leg radiographs measured via the mediCAD 2D planning software (Figure 2).

Microsoft Excel 2007 (Microsoft Corporation, Redmond, USA) was used for descriptive analysis (mean ± standard deviation). R version 4.0.2 with ggplot2 version 3.3.3 was used to create histograms and for all hypothesis tests. Group mean angles were compared with two-sided Welch two-sample t-tests for equality of means, and group proportions were compared using chi-squared tests for equal proportions. For all statistical analyses, single knees were treated as independent observations.

## 3. Results

All 562 postoperative full-leg weight-bearing radiographs could be included in the analysis according to the above-mentioned quality criteria. Mean age at time of surgery in the CIM group was 69.4 ± 10.31 years (range 24–89 years) with a gender distribution of 149 male and 134 female patients. Mean age at time of surgery in the OTS group was 71.7 ± 10.43 years (range 35–92 years) with a gender distribution of 105 male and 174 female patients. In all, 8.5% (24/283) and 5.3% (15/279) of patients had prior surgery on the affected knee in the CIM and OTS group, respectively. Baseline characteristics are shown in Table 1.

### 3.1. Rotational Correction

Calculated rotation in coronal full-leg weight-bearing radiographs in the CIM group ranged from −32.05° internal to 22.57° external rotation of the leg (mean −3.56°, SD 9.65°). Rotation in the OTS group ranged from −1.51° to 23.49° (mean −5.29°, SD 9.10°). Derived correctional factors for HKA ranged from −2.23° varus to 1.57° valgus correction (mean −0.25°, SD 0.67°) in the CIM, and −2.20° to 1.64° correction (mean −0.37°, SD 0.63°) in the OTS group, respectively.

### 3.2. Coronal Alignment

The postoperative radiologically measured corrected and uncorrected HKAs with SD in all 562 patients who underwent TKA are displayed in Table 2.

**Figure 3:**
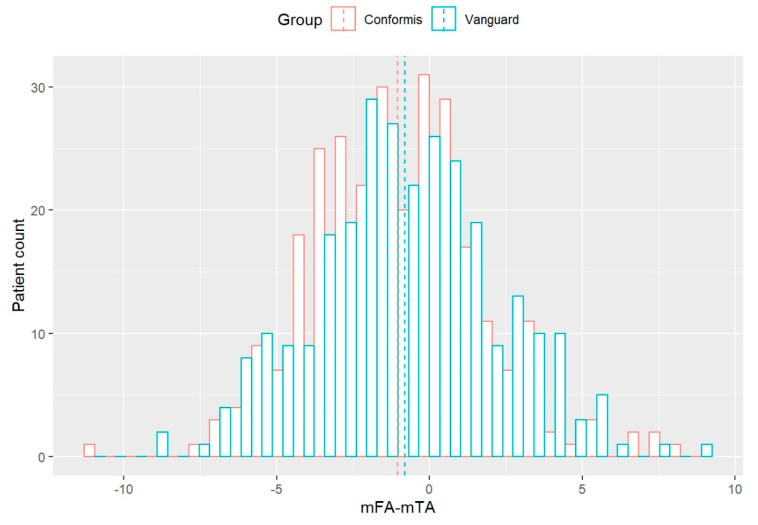
Distribution of corrected postoperative HKA angle in CIM (ConforMIS) and OTS (Vanguard) groups (0 on x-axis corresponds to 180°, dotted lines indicate group mean).

Maximum varus and valgus HKAs were 171.2° (171.2° corrected) and 190.1° (189.2° corrected) in the OTS group and 168.6° (169.3° corrected) and 187.7° (188.21° corrected) in the CIM group, respectively. The distribution of corrected HKAs in both groups is shown in Figure 3. Outliers, outside the 180° ± 3° target zone, were 32.9% in both implant groups (93/283 CIM group; 92/279 OTS group) with a trend toward varus alignment in both groups (CIM group: 71/283 varus; OTS group: 62/279 varus).

The Welch two-sample test for mean corrected HKA between both groups showed no significance, with *p* = 0.34. Further analysis for corrected HKA range +/−1° and +/−3° degrees showed no significant differences between the OTS and CIM group, with p-values *p* = 0.56 and *p* = 1.00, respectively.

## 4. Discussion

In this study, analysis of the up-to-now largest cohort of postoperative coronal leg alignment after implantation of CIM TKA using patient-specific cutting guides and OTS TKA implanted with conventional instrumentation showed equally satisfying results in restoring the HKA angle toward neutral alignment.

To improve surgical technique toward better postoperative leg alignment, computer-aided surgery as well as patient-specific instruments and implants have been developed, especially while conventional techniques using intramedullary guides show high liability to failure due to anatomic variability or surgical error [12]. Although there were no significant differences between leg alignments in both of our groups, we noticed a lower scattering range of leg axis in the CIM group. The rate of outliers in both groups (32.9% with more than ± 3° deviation) was in line with the rates described in other studies [13,14,15,16]. As postoperative leg malalignment and malpositioning of the implant are known to have a high impact on overall outcome and survivorship of TKA [3,13], it is of paramount interest to restore these entities precisely. Whilst computer-aided surgery proved to be superior in restoring leg axis than conventional techniques [17], patient-specific instruments such as cutting guides showed no improvement [18]. Even though patient-specific surgery in TKA is relatively well studied, comparison of CIM and OTS implants and their restoration of leg axis is scarce. Arbab et al. [9] showed no significant difference in pre- and postoperative leg axis change between conventional and patient-specific implants but noticed a trend toward fewer outliers in their CIM group. Steinert et al. [8] detected proper fitting and positioning of the patient-specific implant and a good restoration of leg axis toward neutral alignment. In both studies, coronal full-leg weight-bearing radiographs were used to determine the postoperative leg axis. Because of its complex provision and high liability to failure especially in malrotation [19,20], this radiograph shows a high variability in its reproducibility and therefore in determination of the leg axis. Further, weight-bearing full-leg radiographs are costly and expose the patient’s pelvis to ionizing radiation, which makes correct analysis of the radiographs even more important to reduce recurrent imaging. Various studies have shown alternatives for measuring the long leg axis, but long limb radiographs remain the gold standard [9,20,21]. To further exceed the analyzability of these radiographs, Maderbacher et al. [10,11] published a formula to predict knee rotation via tibio-fibular overlap and to calculate the influence of rotation on the measured alignment parameters. However, this method is limited by the uncertainty of knee flexion during the radiograph, which is common in the early postoperative long-leg radiograph due to painful or mechanical extension deficits. Nevertheless, surgeons should be aware of this method when regularly assessing postoperative long leg radiographs after TKA to prevent incorrect measurement.

The strengths of this study are that it is the largest analysis of custom TKA implants on leg axis and that it considers the rotation in all radiographs as well as its influence on coronal leg alignment. However, we did not take a possible extension deficit after surgery into account. Although full extension of the operated knee was a benchmark for postoperative long leg radiograph in our setting, a bias due to flexion of the knee during X-ray cannot be excluded. Furthermore, due to the retrospective nature of this comparative analysis, a bias for implant selection cannot be excluded. Lastly, we only assessed the ConforMIS iTotal™ CR G2 CIM, and our findings might not be transferable to other patient-specific customized implants.

## 5. Conclusions

TKA using patient-specific cutting guides and implants and OTS TKA implanted with conventional instrumentation resulted in equally satisfying restoration of the coronal leg alignment. When using coronal full-leg weight-bearing radiographs to assess the postoperative leg axis, the modifiers through rotational correction should be taken into account.

## Figures and Tables

**Table 1:** Baseline characteristics.

Variable	CIM (iTotal CR G2)	OTS (Vanguard CR)
Age (years) (mean (SD))	69.5 (10.3)	71.7 (10.4)
Gender		
male	149	105
female	134	174
Side of Surgery		
left	90	115
right	108	127
both	85	37
Previous operation on affected leg (%)	24 (8.5%)	15 (5.3%)

**Table 2:** Postoperative uncorrected and corrected mean HKA ± SD after iTotal™ CR G2 and Vanguard™ CR implantation.

	iTotal™ CR G2 (*n* = 283)	Vanguard™ CR (*n* = 279)
HKA uncorrected	179.2° ± 2.9°	179.6° ± 3.1°
HKA corrected	179.0° ± 2.8°	179.2° ± 3.1°

## Data Availability

The datasets used and/or analyzed during the current study are available from the corresponding author on reasonable request.
